# Health Literacy and Global Cognitive Function Predict E-Mail but Not Internet Use in Heart Failure Patients

**DOI:** 10.1155/2013/507910

**Published:** 2013-10-24

**Authors:** Jared P. Schprechman, Emily C. Gathright, Carly M. Goldstein, Kate A. Guerini, Mary A. Dolansky, Joseph Redle, Joel W. Hughes

**Affiliations:** ^1^Department of Biology, University of Akron, 302 East Buchtel Avenue, Akron, OH 44304, USA; ^2^Department of Psychology, Kent State University, P.O. Box 5190, Kent, OH 44224, USA; ^3^Summa Health System, 525 East Market Street, Akron, OH 44309, USA; ^4^Frances Payne Bolton School of Nursing, Case Western Reserve University, 10900 Euclid Avenue, Cleveland, OH 44106, USA

## Abstract

*Background*. The internet offers a potential for improving patient knowledge, and e-mail may be used in patient communication with providers. However, barriers to internet and e-mail use, such as low health literacy and cognitive impairment, may prevent patients from using technological resources. *Purpose*. We investigated whether health literacy, heart failure knowledge, and cognitive function were related to internet and e-mail use in older adults with heart failure (HF). *Methods*. Older adults (*N* = 119) with heart failure (69.84 ± 9.09 years) completed measures of health literacy, heart failure knowledge, cognitive functioning, and internet use in a cross-sectional study. *Results*. Internet and e-mail use were reported in 78.2% and 71.4% of this sample of patients with HF, respectively. Controlling for age and education, logistic regression analyses indicated that higher health literacy predicted e-mail (*P* < .05) but not internet use. Global cognitive function predicted e-mail (*P* < .05) but not internet use. Only 45% used the Internet to obtain information on HF and internet use was not associated with greater HF knowledge. *Conclusions*. The majority of HF patients use the internet and e-mail, but poor health literacy and cognitive impairment may prevent some patients from accessing these resources. Future studies that examine specific internet and email interventions to increase HF knowledge are needed.

## 1. Introduction

 Healthcare is experiencing a push towards the increasing use of electronic communication. The Health Information Technology for Economic and Clinical Health Act enables providers to receive incentive payments for transitioning to electronic medical records [1]. Allowing patient access to medical records electronically offers the potential to improve health outcomes [2, 3]. For example, electronic portals enable patients to securely communicate with their physician, request medication refills, and view lab results [4]. Electronic resources such as patient portals may even assist patients in disease self-management by tracking refill history and changes in health status. However, adoption has been limited. Although growing in popularity, only 7% of Americans utilize patient portals [5].

 However, internet use among older adults is increasing. In 2005, approximately 30% of adults aged 65 and older reported ever using internet or e-mail, and only 46% of those who used the internet reported daily internet use [6]. More recent estimates suggest that 53% of American adults aged 65 and older report internet or e-mail use and that 70% of those regularly use the internet [7]. As internet use increases, older adults will be better suited to take advantage of electronic resources for healthcare purposes. However, previous estimates of internet use in older adults have not focused on individuals with chronic diseases such as heart failure. As patient portals increase in popularity, patients with chronic conditions requiring considerable symptom monitoring, such as heart failure, may be more likely to access electronic resources for disease self-care. These electronic resources may provide the tools needed to reduce the burden of extensive self-management.

The prevalence of internet and email use among patients with heart failure is not currently known, and heart failure patients may have barriers limiting their ability to successfully navigate electronic resources and apply information. Barriers known to be prevalent in heart failure patients include low health literacy, poor heart failure self-management knowledge, and cognitive impairment. These barriers are associated with decreased compliance [8], increased rehospitalization [9], and increased mortality [10, 11]. Low health literacy is associated with poorer condition-related knowledge [12–14] including worse heart failure knowledge [15]. Adults with limited health literacy struggle to search for pertinent health information on the internet [16]. Heart failure patients with cognitive impairment may also have difficulty navigating the internet. Although cognitive impairment is prevalent in heart failure [17–19], no studies have explored whether cognitive impairment impacts electronic resource use and electronic communication in heart failure patients. Finally, whether patients who use the internet and e-mail have used these resources to gain disease-specific knowledge has not been investigated.

 Despite increasing internet use among older adults, it is unclear whether internet and e-mail use among heart failure patients are influenced by health literacy or cognitive impairment and whether internet use relates to increased heart failure knowledge. The current study sought to investigate patterns of electronic resource use among older adults with heart failure and whether heart failure patients who report internet and e-mail use have higher health literacy and less cognitive impairment than heart failure patients who do not use internet or e-mail. Additionally, the present study explored whether heart failure patients who use the internet demonstrate more heart failure-related knowledge than individuals who do not use the internet.

## 2. Methods

### 2.1. Participants

The sample consisted of 119 English-speaking older adults with systolic heart failure who were participating in an NIH-funded study examining cognitive function and self-management in older adults with heart failure. The participants were community-dwelling adults living independently, often with a spouse. Participants were 50 to 85 years of age diagnosed as New York Heart Association (NYHA) heart failure class II or III with documented left ventricular ejection fraction less than 40% confirmed by a medical chart review. Participants were excluded if they had untreated sleep apnea, renal failure requiring dialysis, developmental disability impacting functions of daily living, history of substance abuse, neurological disorder (e.g., dementia, stroke), psychotic disorder (e.g., schizophrenia), terminal illness, CABG surgery within the previous 3 months, or head injury with greater than 10 minutes loss of consciousness according to their self-report. See Table 1 for participant demographics.

### 2.2. Procedures

At baseline, participants completed demographic, medical, and psychosocial self-report measures. A brief neuropsychological battery was administered to assess multiple domains of cognitive functioning, including tests of global cognitive function, executive function, attention, and memory.

### 2.3. Measures

*Heart Failure Connectivity.* The Heart Failure Connectivity Questionnaire was created for this study to measure computer and internet use and patterns. This questionnaire was developed by adapting questions from a survey in the Kaiser Family Foundation 2005 Report *e-Health and the Elderly: How Seniors Use the Internet for Health Information *[6]. Fifteen questions were taken from the survey and altered to apply to heart failure patients. The questions assessed the following: internet access and use, e-mail use, internet-related communication with healthcare professionals, reasons for not using a computer or internet, and heart-failure-related internet use.

*METER. *Health literacy was assessed using the Medical Term Recognition Test (METER) [20]. METER scores range from 0 to 40 with higher scores reflecting higher health literacy. Scores from 0 to 20 represent low health literacy. Scores from 21 to 34 represent marginal health literacy. Scores from 35 to 40 represent functional health literacy. The METER demonstrates high internal consistency (i.e., Cronbach's alpha = 0.93). 

*Dutch Heart Failure Knowledge Scale.* The Dutch Heart Failure Knowledge Scale was used to assess general heart failure knowledge as well as knowledge related to symptoms, symptom recognition, and treatment (e.g., diet and fluid restriction) [21]. Scores range from 0 to 15, with higher scores indicating more heart failure knowledge. 

*3MS.* The Modified Minimental State Examination is a short screening measure of global cognitive function, tapping aspects of memory, spatial abilities, and attention [22]. The 3MS includes a variety of short tasks such as letter sequencing, animal naming, mental reversal, and temporal orientation. This test takes approximately 7 minutes to complete and is sensitive to a range of cognitive impairments including Alzheimer's disease and other forms of dementia. Scores below 90 indicated cognitive impairment.

### 2.4. Analytic Strategy

Participant e-mail and internet use were described using descriptive statistics (%). Logistic regression was conducted to examine the relationship between health literacy and cognitive function and the dichotomous variables of internet use (0 = no internet use; 1 = internet use) and e-mail use (0 = no e-mail use; 1 = e-mail use). Analysis of covariance (ANCOVA) was used to determine whether heart failure knowledge differed between internet users and nonusers. Age and education were included as covariates. Statistical analyses were performed using SPSS for windows (version 20).

## 3. Results

 Sample characteristics are presented in Table 1. Most participants reported internet access at home or work (82.4%), using the internet (78.2%) and using e-mail (71.4%). 23.5% of the sample were cognitively impaired. Few participants reported communication with a healthcare provider via the internet. Forty-five percent of the sample reported looking up heart failure on the internet (see Table 2). Most participants demonstrated “functional” health literacy (see Table 3). With regard to the Dutch Heart Failure Knowledge Scale, 79.8% of the patients had scores in the 11–15 range, and 20.2% of the patients had lower to marginal scores in the 7–10 range.

### 3.1. Health Literacy

Two separate logistic regression analyses were conducted to investigate whether METER scores predicted internet use and e-mail use after controlling for age and education. The results of the logistic regression for internet and e-mail use are presented in Tables 4 and 5, respectively. Results of the analysis of e-mail use indicated that individuals with higher health literacy were more likely to report e-mail use (*B* = .08, *P* < .05). METER scores did not predict internet use (*P* = .06).

### 3.2. Cognitive Function

Controlling for age and education, logistic regression indicated that individuals with higher 3MS scores were more likely to report e-mail use (*B* = .09, *P* < .05). The results are presented in Table 6. 3MS scores did not predict internet use (*P* = .23). 

### 3.3. Is Internet Use Related to Heart Failure Knowledge?

ANCOVA between internet use and heart failure knowledge, controlling for age and education, revealed no effect of internet use on the Dutch Heart Failure Knowledge Survey, *F*(1,116) = .089, *P* = .77. Scores were similar in participants who reported internet use (*M* = 12.24, SD = 1.89) and those who reported no internet use (*M* = 11.92, SD = 2.40). 

## 4. Discussion

 Here we report that the majority of heart failure patients surveyed have internet access and use both the internet and e-mail. However, higher health literacy and better cognitive function predicted e-mail use but not internet use. Eventually, internet use may become ubiquitous among heart failure patients, which would limit the ability of patient characteristics to predict use. Of course, our patients were not randomly selected and may not be representative of all patients with heart failure. However, they were recruited from two large medical centers in the Midwest and were typically over 65 years of age. What was striking was the contrast between access and use of the internet and e-mail for heart failure-related purposes; few patients reported having discussed the use of these resources with their physicians, and less than half reported using the internet to find information on heart failure. 

Although there is little literature on use of the internet and e-mail by patients with heart failure, use of these resources will only increase and may hold promise for educating patients regarding heart failure and facilitating communication with healthcare providers. For example, a randomized trial of providing an online medical record to patients with heart failure reported that patients were able to use the system and that self-reported adherence to medications improved [23]. Furthermore, the American College of Cardiology has created a website (https://www.cardiosmart.org/) for patient education that includes many conditions including heart failure. Thus, thought leaders are anticipating a need for internet-based resources for patient use. Supporting effective use of available resources is important given that patients are expected to appropriately manage their health and respond to changes in symptoms [24]. 

In this sample, heart failure knowledge did not differ between internet users and individuals who do not use the internet. Many medical websites exist to provide heart failure-related information, but less than half (45.4%) of the sample reported having looked up heart failure on the internet. Whether information obtained on the internet can help to address poor self-care requires further study. Toward that end, a review of information regarding heart failure on the internet suggested that many websites provide primarily biomedical information as opposed to information designed to be useful to patients and recommended that healthcare providers carefully consider the content of websites prior to recommending them to patients [25]. We are unaware of any trials of internet-delivered patient education for heart failure. 

However, there are many cases where the patients themselves are not solely in charge of their medical needs. It may be a family member, friend, or even a nurse who is in charge of making sure they take their medication or even bathing them. The internet could be a reliable tool for these caregivers if they have questions about the patient's medications or notice some unusual symptoms. In fact, Fox and Brenner of the Pew Research Center [26] stated that “eight in ten caregivers (79%) have access to the internet. Of those, 88% look online for health information” (page 2). They also found that some of searches that these caregivers made on the internet were for reviews of drugs, treatments, doctor, and even hospital facility ratings [26]. This Pew Report reflects that the internet can be a reliable tool for health information, even if it is not the patients themselves directly going online.

### 4.1. Limitations

Convenience sampling may limit the generalizability of these findings, and we did not intend for this to be an epidemiological investigation of rates of internet and e-mail use. Rates of use of electronic resources change rapidly, and we suspect that increasing numbers of patients with heart failure will be using the internet and e-mail. Although the Heart Failure Connectivity Questionnaire was a simple survey, the reliability is admittedly not known and the questions do not lend themselves to evaluation of internal consistency as they do not total up to create a score. Furthermore, the small sample may have limited power to find associations. 

## 5. Conclusions

In sum, the current findings suggest that many heart failure patients can access and use the internet and that higher health literacy and cognitive functioning predict e-mail use, although this sample generally had functional health literacy and dementia was an exclusion criteria. Finally, less than half of the participants used the internet to obtain heart failure knowledge and use of the internet was not related to more heart failure knowledge when compared with individuals who did not report internet use. Electronic health educational resources, including patient portals and medical websites, may represent an untapped resource for bridging the gap between the healthcare team and patient self-management. Interventions using the internet should capture usage statistics to demonstrate an adequate “dose,” as mere access and availability does not ensure use and patient learning. Future studies are needed to investigate the use of health care portal use among heart failure patients and methods to enhance their use.

## Figures and Tables

**Table 1:** Demographic characteristics (*n* = 124).

	*N* (%)	Mean (SD)
Age (years)		69.85 (9.20)
Sex		
Male	84 (70.6)	
Female	35 (29.4)	
Race		
Caucasian	101 (84.9)	
Non-Caucasian	18 (15.1)	
Education level		
8th grade or less	1 (0.8)	
9th to 11th	8 (6.7)	
High school	30 (25.2)	
Technical or trade	17 (14.3)	
Some college	29 (24.4)	
Bachelor's degree	20 (16.8)	
Master's degree	14 (11.8)	
Employment status		
Retired	79 (66.4)	
Retired but work part time	22 (18.5)	
Retired but work full time	2 (1.7)	
Work part time	5 (4.2)	
Work full time	10 (8.4)	
Homemaker	1 (0.8)	
Marital status		
Never married	7 (5.9)	
Married	79 (66.4)	
Widowed	12 (10.1)	
Separated	2 (1.7)	
Divorced	19 (16.0)	
Current living arrangements		
Live alone	21 (17.6)	
Live w/a spouse	78 (65.5)	
Live w/a friend permanently	3 (2.5)	
Live w/a family member temporarily	4 (3.4)	
Live w/a family member permanently	13 (10.9)	

**Table 2:** How connected are heart failure patients?

	*N* (%)
Has a doctor ever asked if you have internet access?	28 (23.5)
Have you ever communicated with a doctor or another provider through e-mail?	16 (13.4)
Has a doctor ever recommended a particular health/medical website?	11 (9.2)
Have you ever looked up information about heart failure on the internet?	54 (45.4)

**Table 3:** METER scores.

	*N* (%)
Low health literacy (0–20)	5 (4.2)
Marginal health literacy (21–34)	17 (14.3)
Functional health literacy (35–40)	97 (81.5)

**Table 4:** Logistic regression analysis predicting internet use from age, education, and health literacy (METER).

	*B *	SE *B *	*e* ^*b*^	95% C.I. for *e* ^*b*^
Age	−.03	.03	.97	.92–1.02
Education	.38*	.16	1.46	1.06–2.01
METER	.07	.04	1.08	.99–1.16
Constant	−1.07			

*χ* ^2^	10.92*			
df	3			

*P < .05.

**Table 5:** Logistic regression analysis predicting e-mail use from age, education, and health literacy (METER).

	*B *	SE *B *	*e* ^*b*^	95% C.I. for *e* ^*b*^
Age	−.03	.02	.97	.92–1.01
Education	.45**	.16	1.56	1.16–2.12
METER	.08*	.04	1.09	1.01–1.17
Constant	−2.10			

*χ* ^2^	16.27**			
df	3			

*P < .05, **P < .01.

**Table 6:** Logistic regression analysis predicting e-mail use from age, education, and cognitive function (3MS).

	*B *	SE *B *	*e* ^*b*^	95% C.I. for *e* ^*b*^
Age	−.02	.03	.98	.94–1.03
Education	.35*	.16	1.42	1.04–1.95
3MS	.09*	.04	1.09	1.00–1.19
Constant	−7.57			

*χ* ^2^	15.20***			
df	3			

*P < .05, ***P < .001.

Note: 3MS Modified Minimental State.
